# Comparison of adsorption affinity of polyacrylic acid for surfaces of mixed silica–alumina

**DOI:** 10.1007/s00396-013-3103-x

**Published:** 2013-11-23

**Authors:** Małgorzata Wiśniewska, Teresa Urban, Elżbieta Grządka, Vladimir I. Zarko, Vladimir M. Gun’ko

**Affiliations:** 1 Department of Radiochemistry and Colloid Chemistry, Faculty of Chemistry, Maria Curie Sklodowska University, Maria Curie Sklodowska Sq. 3, 20-031 Lublin, Poland; 2 National Academy of Sciences of Ukraine, Institute of Surface Chemistry, 17 General Naumov Street, 03164 Kiev, Ukraine

**Keywords:** Polyacrylic acid, Mixed silica–alumina, Polymer conformation, Adsorption, Zeta potential

## Abstract

The influence of solution pH (in the range 3–9) on the adsorption of polyacrylic acid (PAA) on the mixed silica–alumina surface (SA-3: SiO_2_ 97 %–Al_2_O_3_ 3 % and SA-96: SiO_2_ 4 %–Al_2_O_3_ 96 %) was investigated. The following methods were applied in experiments: spectrophotometry, viscosimetry, potentiometric titration, and microelectrophoresis, which enable determination of adsorbed amount of the polymer, thickness of its adsorption layers, surface charge density, and zeta potential of solid particles in the presence and absence of PAA, respectively. The obtained results indicate that rise of solution pH causes the decrease of PAA adsorption and the increase of its adsorption layer thickness on surfaces of both solids. Moreover, significantly higher adsorption of polyacrylic acid was obtained on the SA-96 surface. This is a result of more favorable electrostatic interactions occurring between the adsorbing polymer chains and the SA-96 surface and formation of a greater number of adsorbate-adsorbent connections through hydrogen bridges.

## Introduction

The adsorption process of various substances (low molecular and macromolecular) [1–4] is very important for many practical applications. The polymeric compounds turned out to be very effective in the role of substances used to control colloidal systems stability. The specific conformation of macromolecular chains adsorbed on the surface of colloidal particle is essential for stabilization or destabilization of such systems [5–8]. The research concerning the adsorption of polymeric substances at the solid–liquid interface is very crucial due to wide usage of such systems in many fields of human activity. The most important of them are production of cosmetics, pharmaceuticals, paint, paper, food processing, drinking water purification, wastewater treatment, flotation, mineral processing, agriculture (reduction of cultivated soil erosion), and oil recovery [9–16].

Such huge demand for efficient stabilizers and flocculants of industrial colloidal suspensions makes that basic studies of the adsorption mechanism of polymers on the solid surface, which directly affects the stability of suspension in the presence of macromolecular compound, is very important.

In order to determine the mechanism of the processes taking place during the contact of the polymer solution with a solid surface, there must be taken into account the kind and structure of the macromolecular compound, the surface properties of metal oxide, as well as the type of interactions taking place between them. The precise characteristics of the conformation of adsorbed macromolecules and the impact of various factors on final conformation of adsorbed macromolecules can provide information on the structure of the polymer adsorption layer. This can be done based on the experimentally determined parameters, of which the most important are the amount of adsorbed polymer, its adsorption layer thickness, surface charge, and zeta potential of solid particles in the presence of polymer. The specific placement of the adsorbed polymer chains on the solid surface depends, among others, on the molecular weight of the polymer, its kind (anionic, cationic, and nonionic), concentration, and type of surface groups of the metal oxide, solution pH, and temperature.

Thus, the aim of this work is to determine the adsorption mechanism of anionic polyacrylic acid (PAA) on the mixed silica–alumina surface. As mentioned above, the conformation of the adsorbed polymer chain (especially ionic) depends on many factors; the influence of three of them (solution pH, polymer molecular weight, and metal oxide type) was investigated. Two mixed oxides, differing in chemical composition, were applied in the experiments: SA-3: SiO_2_ 97 %–Al_2_O_3_ 3 % and SA-96: SiO_2_ 4 %–Al_2_O_3_ 96 %. Such small addition of another oxide (of a few percent) to the basic oxide leads to some modification of the solid surface properties, which affects the changes in concentration of surface active groups and the values of the adsorbent pH_pzc_ (point of zero charge) and pH_iep_ (isoelectric point).

Mixed oxides are widely used as components of ceramics, as catalysts for organic synthesis, adsorbents for various gasses, and toxic ions [17–20].

The performed studies are of fundamental research character; however, the obtained results have important practical aspect. Determination of the stability mechanism of a solid–polymer solution system must be preceded by measurements that lead to characterize the conformation of the polymer chains adsorbed on the adsorbent surface. For this reason, the presented studies very well fit in this scientific trend. Our previous research indicated that the polyacrylic acid presence in the systems containing both mixed oxides (SA-3 and SA-96) has pronounced impact on suspension stability (both stabilizing and destabilizing, depending on solution pH and polymer molecular weight) [21, 22].

## Experimental

The samples of mixed silica–alumina were used in the study (pilot plant in the Chuiko Institute of Surface Chemistry, Kalush, Ukraine). Applied oxides were prepared using chemical vapor deposition method [23]. The physicochemical characteristics of these adsorbents and their symbols are placed in Table 1. The BET surface area and the mean pore diameter were determined by the low-temperature nitrogen adsorption–desorption isotherm method (Micrometritics ASAP 2405 analyzer). The mean grain sizes of applied solids were obtained using photon correlation spectroscopy (Zetasizer 3000, Malvern Instruments).

**Table 1:** Physicochemical characteristic of adsorbents

Symbol	Chemical composition	BET specific surface area [m^2^/g]	Mean pore diameter [nm]	Mean grain size [nm]
SA-3	SiO_2_ (97 %) Al_2_O_3_ (3 %)	302	7.7	258
SA-96	SiO_2_ (4 %) Al_2_O_3_ (96 %)	75	7.4	176

Polyacrylic acid (Fluka) with the weight average molecular weights 2,000, 100,000, and 240,000 was used in the experiments. The functional groups of PAA chains are carboxyl (–COOH) ones. As a result of the pH increase, the dissociation of functional groups occurs. The pKa of the PAA is 4.5 [24], which means that at pH below 4.5 functional groups in the polymer chains are predominantly undissociated (at pH 3, the degree of these groups dissociation (*α*) is equal to 0.03). At pH 4.5, the number of –COOH groups is the same as –COO^−^ ones (*α* = 0.5). As the pH rises above 4.5° of polyelectrolyte dissociation increases rapidly; at pH 6, it is equal to 0.97 and at pH 9 reaches a value close to 1 [25].

All measurements were carried out in the presence of NaCl solution (1 · 10^−2^mol dm^3^) which was used as the supporting electrolyte. Adsorption, viscosity, and electrokinetic experiments were performed at the solution pH 3, 6, and 9 at 25 °C. The appropriate pH values of the investigated systems were adjusted using a pH meter PHM 240 (Radiometer) with the accuracy ±0.1.

The adsorbed amounts of the polymer were determined by the static method using the following polymer concentrations: 10, 20, 30, 50, 70, 100, and 200 ppm. Before the solid addition, the appropriate solution pH (3, 6, or 9) was adjusted with HCl and NaOH solutions in each sample (with a volume of 10 cm^3^). Then, 0.04 g of SA-3 (or 0.015 g of SA-96) was added to each polymer solution, and suspension pH was checked and adjusted. The prepared suspensions were shaken for about 24 h in water bath (OLS 200, Grant Inst.), and then the solution pH was checked again. Afterwards, the solid was centrifuged and 5 cm^3^ of clear solution were taken for further analysis. The polymer concentration in each sample was determined using hyamine [26] The turbidity of solutions after the hyamine addition was measured after 15 min at a wavelength *λ* = 500 nm with a UV/VIS spectrophotometer (Carry 100 Bio, Varian). The amount of adsorbed polymer was determined from the difference in the polymer concentration in the solution before and after the adsorption process using the previously prepared calibration curve (dependency of absorbance of polymer solution versus its concentration).

The thickness of the polymer adsorption layers (*δ*) was determined from the viscosity measurements [27] using a CVO 50 rheometer (Bohlin Instruments). For this purpose, the following dependencies were used:

1 $$ \delta =r\left[{\left(\frac{\varphi_{{}_p}}{\varphi_0}\right)}^{1/3}-1\right] $$

Where *r* is the radius of the solid particle, *ϕ*_0_ is the volume fraction of the solid without polymer, and *ϕ*_*p*_ is the volume fraction of the solid with polymer.

2 $$ \frac{\eta }{\eta_0}=1+k{\varphi}_0 $$

Where *η* is the viscosity of the suspension, *η*_0_ is the viscosity of the liquid phase, and *k* is the Einstein coefficient.

**Fig. 1:**
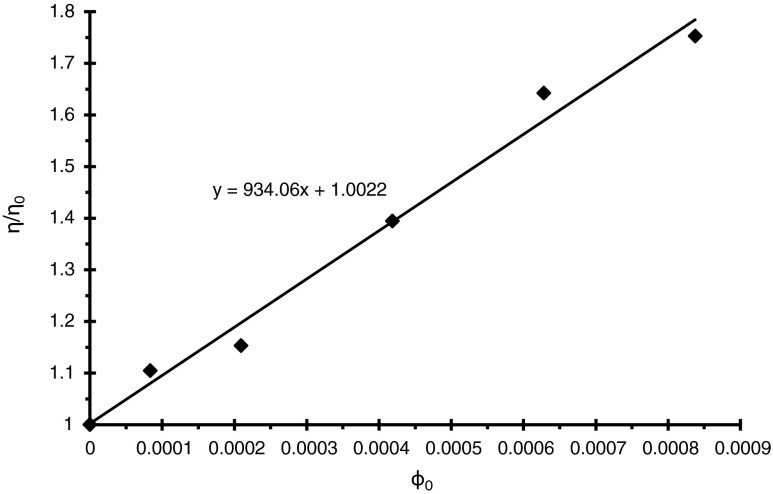
Dependence of SA-3 suspension relative viscosity (*η*/*η*_0_) versus solid volume fraction (*ϕ*_0_)

The values of *ϕ*_*p*_ were obtained from a calibration curve. For this purpose, a few suspensions of mixed oxide corresponded to various volume fractions (*ϕ*_0_) of the solid were prepared in the following way: 0.5, 1, 1.5, 2, and 2.5 g of the solid were added to 40 ml of NaCl solutions. Then, these suspensions were shaken for 24 h in water bath OLS 200 (Grant). After this time, their viscosities (*η*) and viscosities of clear NaCl solution (*η*_0_) were measured. As a result, the *η*/*η*_0_ = f(ϕ_0_) dependence was obtained (straight line). An example of calibration curve obtained for SA-3 system is presented in Fig. 1.

The viscosity measurements in the presence of PAA were made with the volume fraction (*ϕ*_0_) of SA-3 equal to 1.25 · 10^−4^ and SA-96 1.89 · 10^−3^. The value of volume fraction (*ϕ*_p_) corresponding with (*η*/*η*_0_)_p_ ratio in the presence of polymer was read out from the calibration curve. The polymer adsorption causes the increase of volume fraction of dispersed solid. Knowing the radius of the solid particle, the thickness of the adsorbed polymer layer can be calculated using Eq. [1].

The potentiometric titration method was used to determine the surface charge density of the mixed oxide in the absence and presence of PAA. This method is based on the comparison of titration curve of electrolyte with the titration curve of suspension (containing adsorbent) having the same ionic strength (supporting electrolyte concentration). Such comparison of both titration curves allows determination of the pH_pzc_ (point of zero charge, pzc); it is located at the intersection of these two curves. The surface charge density (*σ*_0_) is determined from the difference in the volume of base (*Δ*V) which must be added to bring the pH of suspension and electrolyte to the specified value:

3 $$ {\sigma}_0=\frac{\varDelta V{c}_bF}{ mS} $$

Where *c*_*b*_ is the base concentration, *F* is the Faraday constant, *m* is the solid mass in the suspension, *S* is the specific surface area of the solid.

The experimental setup for these measurements included: Teflon vessel, automatic burette Dosimat 665 (Methrom), thermostat RE204 (Lauda), pH meter 71 pH meter (Beckman), and computer. Special computer program Titr_v3 (author: W. Janusz) collected data and performed surface charge density calculations. SA-3 0.2 g (or 0.3 g of SA-96) was added into the thermostated vessel to 50 cm^3^ of supporting electrolyte solution or polymer solution (C_PAA_ = 100 ppm) in the supporting electrolyte. The suspensions prepared in such a way were titrated with the NaOH solution (1⋅10^-1^ mol/dm^3^) in the pH range 3–10.

The zeta potentials of mixed oxide particles in the presence and absence of PAA were measured with the Zetasizer 3000 laser zetameter (Malvern Instruments). For that purpose, the suspensions containing 0.04 g of SA-3 (or 0.01 g of SA-96) in 500 cm^3^ of the supporting electrolyte or polymer solution (C_PAA_ = 0.1 ppm) were prepared. This suspension was ultrasonicated for 3 min (Ultrasonic Processor XL, Misonix) and divided into six parts. Next, the appropriate pH value (3; 4.5; 5.5; 6.5; 8 and 10) was adjusted in each sample and its potential zeta was measured. The value of the zeta potential was calculated with a suitable computer program using the Smoluchowski equation.

## Results and discussion

**Fig. 2:**
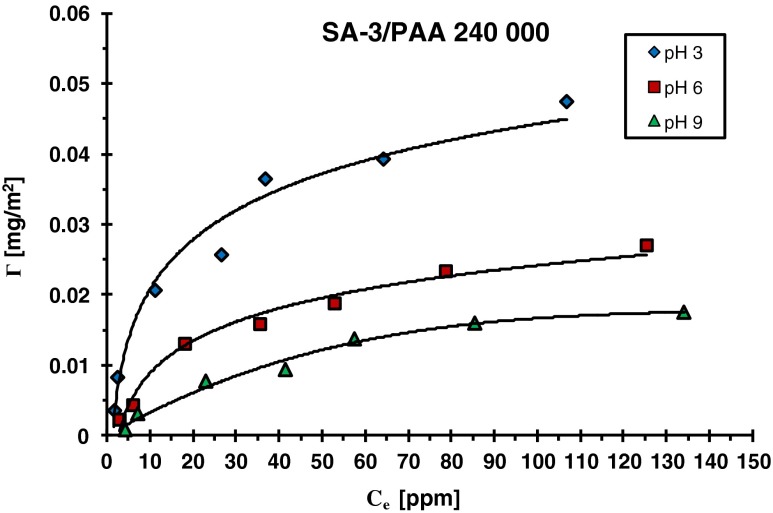
Adsorption isotherms of PAA 240,000 on the SA-3 surface at different solution pH values

**Fig. 3:**
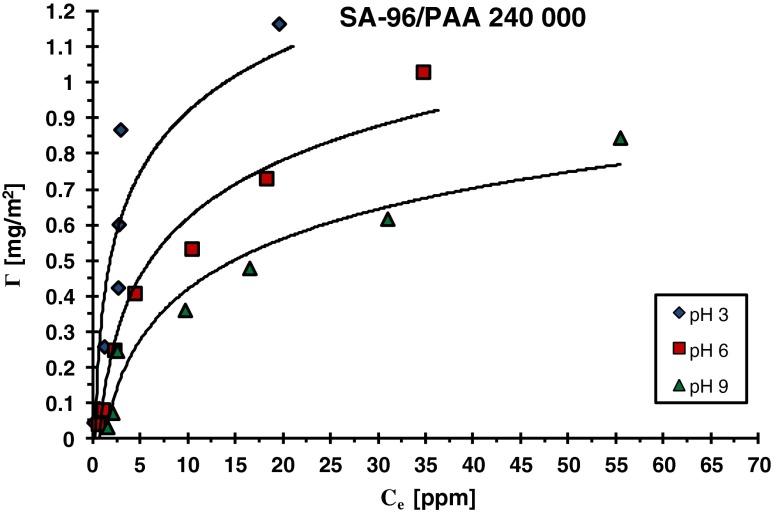
Adsorption isotherms of PAA 240,000 on the SA-96 surface at different solution pH values

Figures 2 and 3 show the adsorption isotherms of polyacrylic acid with molecular weight 240,000 on the surfaces of SA-3 and SA-96 determined at different pH values (i.e., 3, 6, and 9). For the two other molecular weights of PAA (2,000 and 100,000), similar dependencies were obtained. As can be seen in these figures, the solution pH at which the polymer adsorption takes place has a significant influence on its amount. Analysis of presented isotherms leads to the conclusion that PAA adsorption on the mixed oxide surface decreases with the increasing pH of the solution. The pH changes affect the process of carboxyl groups’ dissociation in the polymer chains which result in the changes of ionization of polyacrylic acid macromolecules. The pK of PAA is equal to 4.5; thus, before reaching this pH value, the undissociated carboxyl groups –COOH predominate in the polymer chain over the dissociated ones –COO^−^. At pH 4.5, the number of undissociated carboxyl groups is the same as that of dissociated ones. Further, pH increase above the value 4.5 results in dissociation of the remaining carboxyl groups. At pH 6, PAA macromolecules are already almost completely ionized [degree of dissociation (*α*) is equal to 0.97] [25].

**Fig. 4:**
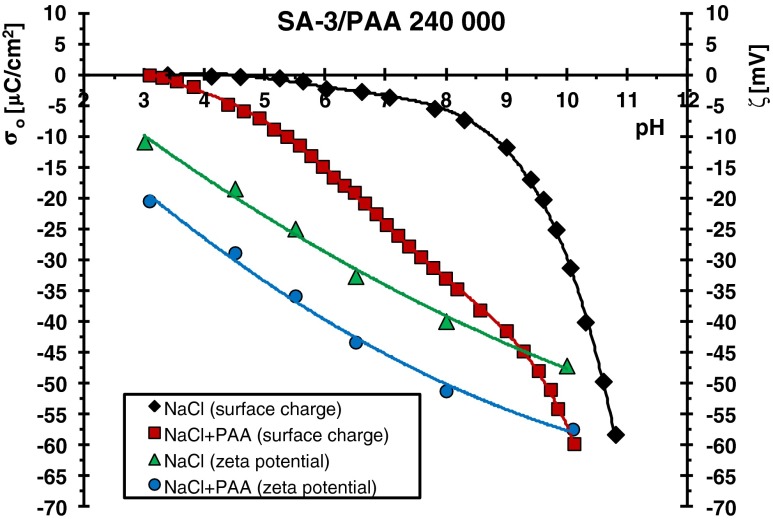
Surface charge density (*σ*_o_) and zeta potential (*ζ*) of SA-3 particles in the absence and presence of PAA 240,000

**Fig. 5:**
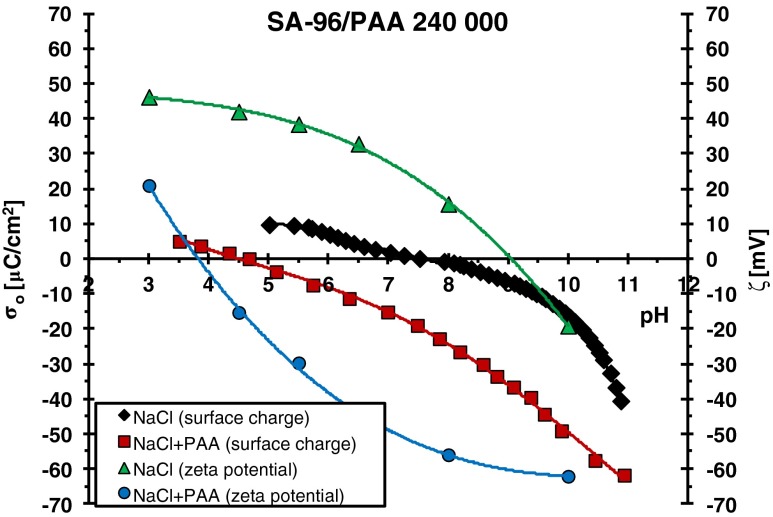
Surface charge density (*σ*_o_) and zeta potential (*ζ*) of SA-96 particles in the absence and presence of PAA 240,000

On the other hand, the surface charge density of the adsorbents has the crucial effect on the interactions between the adsorbent and the adsorbate. Figures 4 and 5 present, among others, the surface charges of examined oxides as a function of pH. The points of zero charges are at the pH values: 3.4 for SA-3 and 7.6 for SA-96. At these pH values, the total surface charge is equal to zero, which means that the numbers of positively and negatively charged surface groups are the same.

Taking above into consideration, it can be stated that in the case of the SA-3/PAA systems (Figs. 2 and 4), the electrostatic repulsion between the solid surface and the polymer chains occurs almost in the whole examined pH range. For this reason, the polymer adsorption decrease with the pH rise is a result of the increasing repulsion between the dissociating PAA macromolecules and more negative solid surface. Under such conditions, the polymer adsorption on the SA-3 surface undergoes through hydrogen bridges and chemical bonds [28, 29]. It results from the fact that carboxyl groups of the polymer can act both as a donor and an acceptor of protons. Thus, adsorbate–adsorbent bonding can proceed between all types of solid surface groups (negative, positive, and neutral) and undissociated and dissociated PAA functional groups.

A little different situation takes place in the case of the SA-96/PAA systems (Figs. 3 and 5). pH_pzc_ of this oxide is 7.6 and in the pH values below 7.6 attractive interactions between the dissociated carboxyl groups of PAA and the positively charged surface take place. Such adsorbent–adsorbate attractions cause a significant increase of the polymer adsorption at pH 3. Another factor which promotes the adsorption of PAA is its specific conformation. At pH 3, PAA macromolecules contain a small number of –COO^−^ groups (dissociation degree 0.03), and they adsorb on the SA-96 surface in the form of polymeric coils. Such a structure of the adsorbed chains leads to the formation of densely packed polymer films. As a result, the highest adsorption of polyacrylic acid at pH 3 is observed. More and more extended conformation of adsorbing polymer chains with the increasing pH and the weakening attraction gradually passing into repulsion leads to pronounced reduction in polymer adsorption. However, despite unfavorable electrostatic repulsion at pH >7.6, PAA adsorption takes place. This fact proves that specific interactions, which include hydrogen and chemical bonds, are responsible for the binding of polymer macromolecules with the solid surface.

Electrostatic repulsion polymer surface occurring practically in the whole range of studied pH in the case of SA-3 causes minimal adsorption of PAA on its surface. Table 2 presents the comparison of adsorbed amounts (Γ) of PAA (with various molecular weights) on the SA-3 and SA-96 surfaces at different solution pH values (obtained for the initial PAA concentration 100 ppm). The adsorbed amounts of polyacrylic acid are several (2 or 3) dozen times higher on the SA-96 surface than those observed on the SA-3 surface. A much greater adsorption affinity of PAA chains to the SA-96 surface is mainly due to the favorable attractive interactions which are present in the system at the pH range below pH_pzc_ (namely at pH <7.6). The electrostatic repulsion between the PAA and SA-96 surface appearing at pH >7.6 is similar to that obtained in the case of the adsorbent SA-3. For both applied mixed oxides, the surface charge density changes in the pH range 7.6 to 9 from −4.5 to −11.9 μC/cm^2^ for SA-3 (Fig. 4) and from 0.12 to −7.1 μC/cm^2^ for SA-96 (Fig. 5). It means that electrostatic repulsion in this pH range has similar strength for both adsorbents. Thus, the adsorption of polymer in this pH range must be determined by the concentration of neutral –MeOH (Me—metal atom: Si or Al) surface groups. The previous study [30] showed that the concentration of these surface groups is several dozen times larger than that of charged ones (positive –MeOH_2_^+^ and negative –MeO^−^). In the case of alumina, the concentration of neutral groups on its surface in the pH range 7–9 is about 120 μC/cm^2^ [31], whereas for silica, it is about a few microcoulombs per cubic centimeter (on average 7 μC/cm^2^) [32].

**Table 2:** Comparison of adsorbed amounts (*Γ*) of PAA on the SA-3 and SA-96 surfaces at different solution pH values for C_PAA_ = 100 ppm

Polymer	SA 3			SA 96		
	pH = 3	pH = 6	pH = 9	pH = 3	pH = 6	pH = 9
PAA 2,000	0.02	0.015	0.013	0.64	0.59	0.51
PAA 100,000	0.03	0.02	0.015	0.78	0.63	0.58
PAA 240,000	0.04	0.03	0.016	0.86	0.73	0.62

Taking this fact into consideration, one can assume that in the case of SA-96 surface, the number of neutral surface groups must be significantly greater in comparison to SA-3. For this reason, despite comparable repulsion between the polymer and the adsorbent in the case of both examined oxides, the adsorbed amount of PAA is considerably greater on the SA-96 surface. This great affinity of PAA chains for the SA-96 surface under such pH conditions probably results from the possibility of formation of a greater number of connections between the polymer segments and –MeOH surface groups (whose concentration must much greater in the case of SA-96).

The confirmation of the PAA adsorption mechanism proposed above is thicknesses of its adsorption layers on the surfaces of studied oxides. The comparison of adsorption layer thicknesses (*δ*) of PAA (with various molecular weights) on the SA-3 and SA-96 surfaces at different solution pH values is presented in Table 3. The analysis of these data indicates that *δ* increases with the pH rise. Moreover, for all examined molecular weights of polyacrylic acid, thicker adsorption layers of PAA are formed on the SA-3 surface.

**Table 3:** Comparison of adsorption layer thicknesses (*δ*) of PAA on the SA-3 and SA-96 surfaces at different solution pH values for C_PAA_ = 100 ppm

Polymer	SA 3			SA 96		
	pH = 3	pH = 6	pH = 9	pH = 3	pH = 6	pH = 9
PAA 2,000	2.8	5.3	9.6	2.0	4.8	7.4
PAA 100,000	3.6	7.4	10.1	3.5	5.1	8.3
PAA 240,000	4.9	9.0	11.7	4.3	7.9	10.0

The increase of thickness of PAA adsorption layers with the increasing pH results from rising dissociation of carboxyl groups in the polymer chains and changes in the sign of surface charge from positive to negative (at pH 3.4 for SA-3 and at pH 7.6 for SA-96). Mutual repulsion between the growing number of dissociated-COO^−^ groups in the PAA macromolecules leads to their straightening as pH rises. On the other hand, the process of developing of the polymer chains is amplified by the weakening attraction of them to the surface and after exceeding the pH_pzc_ by electrostatic repulsion. As a result, the higher the solution pH is, the thicker the polymer adsorption layer is.

Additionally, in the case of SA-3, the thicker PAA adsorption layers are formed in comparison to those obtained for SA-96. The main reason for such behavior is small affinity of polyacrylic acid macromolecules for the SA-3 surface. As a result, a few polymer segments are binding to the active sites on the surface of the adsorbent, so that the conformation of the adsorbed macromolecule is rich in long loop and tail structures. In the case of SA-96, the adsorbed polymer chains have flatter conformation due to the larger number of direct connections of PAA segments with the surface –MeOH groups, which in the case of this solid must be much more. Also of importance is the fact of the presence of electrostatic repulsion between the PAA chains and the SA-3 surface practically in the whole range of studied pH. It affects a significant expansion of adsorbed macromolecules in perpendicular direction to the solid surface, which is reflected in the increase of the thickness of the polymer adsorption layer formed in the SA-3/PAA systems.

As can be seen in Figs. 4 and 5, polyacrylic acid adsorption causes lowering of both oxide surface charge density (*σ*_o_) and zeta potential (*ζ*) of solid particles. Such tendency was observed for both examined mixed oxides and for all used molecular weights of PAA. Considering the fact that the presence of PAA with the highest molecular weight causes the greatest reduction in these two parameters, in this paper, respective results for the mixed oxide/PAA 240,000 systems are presented.

Lowering of the surface charge of both adsorbents in the presence of polyacrylic acid is a result of the two effects. On one hand, the dissociated carboxyl groups of the polymer, which undergo the direct binding to the solid surface cause increase of the solid surface charge (usually anionic compounds adsorption forces the formation of positively charged groups on the surface). On the other hand, only a few polymer segments are binding to the adsorbent surface; the overwhelming majority of them are in the solution building loop and tail structures of adsorbed macromolecules. The presence of ionized carboxyl groups belonging to these segments, located in the Stern layer, leads to decrease of the solid surface charge. This latter effect is predominant over the first one because of a much larger number of segments located in the loops and tails compared to the train structures (formed by the polymer segments which are directly connected with the solid surface). As a consequence, the final result of the polyacid adsorption is reduction of the surface charge of mixed oxide.

Three main reasons are responsible for the changes of zeta potential of solid particles in the presence of polymeric substances. These are (a) the presence of functional groups in polymer chains which are capable of dissociation, (b) the shift of slipping plane from the solid surface due to the formation of the polymer adsorption layer [26], and (c) the blockade of solid active sites by the previously adsorbed polymer chains, as a result, they become unavailable for supporting electrolyte ions. The contributions of these effects influence the final value of zeta potential.

As can be seen in Figs. 4 and 5, the changes of zeta potential of oxide particles in the PAA presence in comparison to that in the polymer absence is considerably greater in the case of the system containing SA-96. The difference of *ζ* value (between that for the system without and with PAA) at a given pH value is even 70 mV for SA-96 (at pH 8), whereas for SA-3, this difference is at the level of 10 or 15 mV. The effect of slipping plane shift is undoubtedly responsible for reduction of zeta potential of solid particles covered with polyacrylic acid for all examined adsorbents. Nevertheless, the significantly greater reduction of zeta potential in the system SA 96/PAA in comparison to SA-3/PAA results from the presence of a much larger number of –COO^−^ groups of polymer chains in the diffusion part of the electrical double layer around the solid particle. Such a high concentration of dissociated carboxyl groups in the slipping plane is a result of large adsorption of PAA on the SA-96 surface and less stretched conformation of the adsorbed macromolecules (in perpendicular direction to the solid surface). In the case of the SA-3/PAA system, the polymer adsorption is small and adsorbed chains conformation is more extended, which makes that the packing of the adsorption layer is low and the content of the –COO^−^ groups in the diffusion layer is also small. It leads to far lower reduction of zeta potential of SA-3 particles in the polyacrylic acid presence in comparison to that obtained for the SA-96/PAA system.

The blockade of solid active sites by the previously adsorbed polymer chains may have an effect on the zeta potential changes. It occurs especially in the situation in which the adsorbed macromolecules form flatter conformation and densely packed adsorption layer, i.e., for the system containing SA-96. In such a case, the supporting electrolyte ions are desorbed from the solid surface by the adsorbing polymer chains and they are removed to the diffusion area, influencing the zeta potential of solid particles.

## Conclusions

Of the two examined mixed oxides (SA-3 and SA-96), significantly greater affinity for the adsorption of anionic polyacrylic acid is exhibited by SA-96. It results from much more favorable electrostatic interactions occurring between the polymer chains and the SA-96 surface (attractive forces in the pH range 3–7.6). As a consequence, the PAA adsorption layer formed on the SA-96 surface is characterized by high packing and the adsorbed macromolecules are flatter conformation (thinner adsorption layer). In the case of the SA-3/PAA system, polymer adsorption is small due to the electrostatic repulsion between the PAA chains and the solid surface occurring practically in the whole range of studied pH (3.4–9). As a result, the polymeric adsorption layer on the SA-3 surface is thicker. The hydrogen bridges are responsible for PAA adsorption on the mixed oxide surface, especially under the conditions of electrostatic adsorbate–adsorbent repulsion. Moreover, the presence of PAA causes lowering of surface charge and zeta potential of solid particles in comparison to the system without polymer. The effect of zeta potential reduction is considerably greater in the case of the SA-96 /PAA suspension, mainly due to high concentration of dissociated carboxyl groups of polymer chains in the slipping plane area around the solid particle.
